# Effects of Wait Times on Treatment Adherence and Clinical Outcomes in Patients With Severe Sleep-Disordered Breathing: A Secondary Analysis of a Noninferiority Randomized Clinical Trial

**DOI:** 10.1001/jamanetworkopen.2020.3088

**Published:** 2020-04-20

**Authors:** Christina S. Thornton, Willis H. Tsai, Maria J. Santana, Erika D. Penz, W. Ward Flemons, Kristin L. Fraser, Patrick J. Hanly, Sachin R. Pendharkar

**Affiliations:** 1 Department of Medicine, Cumming School of Medicine, University of Calgary, Calgary, Alberta, Canada; 2 Department of Community Health Sciences, Cumming School of Medicine, University of Calgary, Calgary, Alberta, Canada; 3 Sleep Centre, Foothills Medical Centre, University of Calgary, Calgary, Alberta, Canada; 4 College of Medicine, University of Saskatchewan, Saskatoon, Saskatchewan, Canada

## Abstract

**Importance:**

Sleep-disordered breathing (SDB) is common and associated with substantial adverse health consequences. Long wait times for SDB care are commonly reported; however, it is unclear whether wait times for care are associated with clinical outcomes.

**Objective:**

To evaluate the association of wait times for care with clinical outcomes for patients with severe SDB.

**Design, Setting, and Participants:**

This study is a secondary analysis of a randomized clinical noninferiority trial comparing management by alternative care practitioners (ACPs) with traditional sleep physician–led care between October 2014 and May 2017. The study took place at Foothills Medical Centre Sleep Centre, a tertiary care multidisciplinary sleep clinic at the University of Calgary. Patients with severe SDB (defined as a respiratory event index ≥30 events per hour during home sleep apnea testing, mean nocturnal oxygen saturation ≤85%, or suspected sleep hypoventilation syndrome) were recruited for the study. Patients were excluded if they were suspected of having a concomitant sleep disorder other than SDB or had previously been treated with positive airway pressure (PAP) therapy for SDB. Data were analyzed from October 2017 to January 2020.

**Main Outcomes and Measures:**

Outcomes were assessed 3 months after treatment initiation with adherence to PAP therapy as the primary outcome. Secondary outcomes included Epworth Sleepiness Scale score, health-related quality of life, and patient satisfaction measured using the Visit-Specific Satisfaction Instrument–9. Multiple regression models were used to assess the associations between wait times and each of the outcomes. *t* tests were used to compare wait times for patients who were adherent to PAP therapy (≥4 hours per night for 70% of nights) with those for nonadherent patients.

**Results:**

One hundred fifty-six patients (112 [71.8%] men; mean [SD] age, 56 [12] years) were included in the analysis. The mean time from referral to initial visit was 88 days (95% CI, 79 to 96 days), and the mean time to treatment was 123 days (95% CI, 112 to 133 days). Shorter wait time to treatment initiation was associated with adherence to PAP therapy (odds ratio, 0.99; 95% CI, 0.98 to 0.99; *P* = .04), greater improvement in Epworth Sleepiness Scale score (mean coefficient, −9.37; 95% CI, −18.51 to −0.24; *P* = .04), and higher Visit-Specific Satisfaction Instrument–9 score (mean coefficient, −0.024; 95% CI, −0.047 to −0.0015; *P* = .04) at 3 months. Compared with nonadherent patients, those who were adherent to treatment waited a mean of 15 fewer days (95% CI, 12 to 19 days) for initial assessment (*P* = .07) and 30 fewer days (95% CI, 23 to 35 days) for treatment initiation (*P* = .008).

**Conclusions and Relevance:**

Earlier initiation of treatment for severe SDB was associated with better PAP adherence and greater improvements in daytime sleepiness and patient satisfaction. These findings suggest that system interventions to improve timely access may modify patient behavior and improve clinical outcomes.

**Trial Registration:**

ClinicalTrials.gov Identifier: NCT02191085

## Key Points

**Question:**

What is the association of wait times with clinical outcomes for patients with sleep-disordered breathing?

**Findings:**

In this secondary analysis of a randomized noninferiority clinical trial that included 156 patients, shorter wait time to treatment initiation was associated with greater adherence to positive airway pressure therapy and improved patient-reported outcomes.

**Meaning:**

These findings suggest that system interventions to improve timely access to sleep-disordered breathing treatment are associated with improved clinical outcomes.

## Teaser

This secondary analysis of a randomized noninferiority clinical trial evaluates the association of wait times for care with clinical outcomes for patients with severe sleep-disordered breathing.

## Introduction

Sleep-disordered breathing (SDB) comprises several entities, including obstructive sleep apnea (OSA), central sleep apnea, and sleep-related hypoventilation.^1^ The most common type of SDB is OSA; in adults aged 30 to 70 years, the estimated prevalence of OSA (defined by an apnea-hypopnea index of at least 5 events per hour as measured by polysomnography [PSG]) is 34% in men and 17% in women.^2,3^ Untreated OSA is associated with increased cardiometabolic risk and excessive sleepiness, leading to poor quality of life, depression, and a higher risk of motor vehicle crashes. Patients with severe OSA (defined as apnea-hypopnea index >30 events per hour) have a 2- to 3-fold increased risk of all-cause mortality compared with the general population.^4^

Long delays for care are a paramount concern in many jurisdictions. Longer wait times for surgical procedures, such as hip repair or cataract surgery, are associated with increased pain and disability.^5^ Conversely, improving timely access to treatment for chronic disease may reduce the risk of related complications. For example, among patients aged 65 years or older with cataracts, those who received repair within 1 year had a lower incidence of hip fracture compared with those who did not.^6^ Patients consider timely access an important and unmet health care need.^7^

Long wait times for diagnosis and treatment of SDB have been observed in many jurisdictions,^8,9,10,11^ In 2004, Flemons et al^8^ evaluated wait times for diagnosis and treatment with continuous positive airway pressure (PAP) across 5 countries: the UK, Belgium, Australia, the US, and Canada.^8^ The spectrum in wait times was highlighted across these countries, ranging from 2 months in Belgium to 36 and 60 months in Canada and the UK, respectively. There were long delays for diagnosis and treatment in Ontario, Canada, highlighting the marked variability associated with geographic location, practice setting, and other factors. In most cases, wait times exceeded published guidelines, which recommend wait time targets for assessment and treatment of SDB on the basis of severity, comorbidity, and daytime symptoms.^10^

In 2011, the Canadian Thoracic Society published clinical practice guidelines on the diagnosis and management of SDB.^12^ Recommendations included that assessment be completed within 4 weeks for urgent cases (suspected severe SDB, patients in safety critical occupations or those with significant comorbidity) and within 6 months for all suspected cases. Despite this recommendation, long wait times for SDB care have been widely reported,^8,9,10,11^ in part because of a shortage of sleep physicians. Delayed care may adversely affect the clinical outcomes and response to treatment of patients with SDB; however, there are limited published data on the nature or extent of this association. The goal of this study was to explore the association of wait times with clinical outcomes, including the response to PAP therapy, for patients with severe SDB.

## Methods

### Study Design

This is a secondary post hoc analysis of a randomized clinical noninferiority trial comparing management of severe SDB using registered respiratory therapists functioning as alternative care practitioners (ACPs) to traditional sleep physician–led care.^9,13^ Ethical approval for this study was obtained by the University of Calgary Conjoint Health Research Ethics board. Written informed consent was obtained from all patients included in the study. This study followed the Consolidated Standards of Reporting Trials (CONSORT) reporting guideline for randomized clinical trials.

### Primary and Secondary Objectives

The primary objective of this analysis was to determine the association of wait times with treatment adherence. The secondary objective was to determine associations of wait times with patient-reported outcomes, including daytime sleepiness, disease-specific health-related quality of life, and patient satisfaction.

### Patient Population

The protocol of the full study has been published previously^13^ and is shown in Supplement 1. Briefly, patients with severe SDB (severe OSA with or without hypoventilation) were recruited at the time of referral to the Foothills Medical Centre Sleep Centre (Calgary, Alberta, Canada). The Sleep Centre is the sole publicly funded level I facility within the Calgary zone, which serves a population of 1.6 million people. Patients were included if they had 1 of the following: severe OSA (respiratory event index [REI] ≥30 events per hour according to home sleep apnea testing), mean nocturnal oxygen saturation (≤85%), or suspected sleep hypoventilation syndrome (an REI of ≥15 events per hour and partial pressure of carbon dioxide ≥45 mm Hg on arterial blood gas). The REI was defined as the number of apnea and hypopnea events per hour of monitoring time on a home sleep apnea test. Patients already receiving supplemental oxygen were included if the airflow channel suggested severe OSA. Patients were excluded if they were suspected to have a concomitant sleep disorder other than SDB, had previously been treated with PAP therapy for SDB, had primary health insurance from outside Alberta, or did not provide consent to participate in the study.

### Study Procedure

Briefly, individuals who met inclusion criteria were randomized in a 1:1 allocation to standard management by a sleep physician or to an ACP-led clinic.^9^ In contrast to traditional physician-led assessment in the standard management arm, ACPs in the latter clinic assessed patients and developed a management plan; a sleep physician reviewed this plan and met the patient briefly at the end of the visit (eFigure in Supplement 2). Follow-up could be delegated to the ACP or sleep physician at the discretion of the initial clinician (ACP or physician), with any additional testing or treatment decisions at the discretion of the primary sleep physician following discussion with the ACP. The ACPs could also refer patients back to the sleep physician at any time for persistent SDB symptoms or management of clinical issues other than SDB. Sleep physicians interpreted all tests and signed all treatment prescriptions.

### Study Outcomes

Treatment adherence was determined by PAP machine download at 3 months and was based on the mean nightly hours of use over the previous 4 weeks; adherence was analyzed as both a continuous variable (hours per night) and as a binary variable defined by use of PAP for 4 or more hours per night on at least 70% of nights.^14^ Wait times were also measured for each patient from time of referral to both the time of initial assessment and time of PAP treatment initiation.

The following questionnaires were administered at baseline and 3 months after initiation of PAP therapy: the Epworth Sleepiness Scale (ESS); general health-related quality of life, measured using the Health-Utilities Index–Mark 3; and disease-specific health-related quality of life, measured using the short-form Sleep Apnea Quality of Life Index. Patient satisfaction was measured at 3 months using the Visit-Specific Satisfaction Instrument–9.^15^

### Statistical Analysis

Descriptive analysis was used to assess baseline patient characteristics. Multiple linear and logistic regression models were constructed to identify possible factors associated with wait times and subsequently to assess associations of wait times with study outcomes. First, exploratory univariate analysis was performed on baseline characteristics to identify possible factors associated with wait times. Multiple linear regression models were then constructed using wait time to treatment as the dependent variable. Independent variables included possible factors identified on univariate analysis, as well as baseline characteristics known (or suspected) to be associated with wait times. To evaluate the possibility that an association between shorter wait times and greater PAP use might reflect a tendency for patients who were motivated to attend clinical appointments to also be adherent to PAP, missed appointments for clinician visits or PSG were included in the analysis. Baseline characteristics associated with increased time to treatment on multivariable analysis were included in subsequent models assessing the effects of wait times on adherence and patient-reported outcomes. Second, exploratory univariate analysis was performed on baseline characteristics to identify possible factors associated with improved adherence. Multiple logistic regression models were constructed using adherence as the dependent variable and baseline characteristics deemed to be associated with either wait times or adherence as independent variables. Similarly, univariate and multiple linear regression models were constructed using patient-reported outcomes (change in ESS score, change in Health-Utilities Index-Mark 3 score, change in Sleep Apnea Quality of Life Index score, and Visit-Specific Satisfaction Instrument–9 score at 3 months) as dependent variables. As in the original study, both intention-to-treat and per-protocol analyses were performed. Multivariable models were adjusted a priori for study group even if there was no association in the univariate model, including interaction terms for wait times and study arm. Two-tailed *t* tests were used to compare wait times for patients who were or were not adherent to PAP therapy (defined as ≥4 hours of use per night for ≥70% of nights^14^). All analyses were performed using Stata MP statistical software version 13.1 (StataCorp) with a significance level set at α = .05. Data are presented as a mean (95% CI) unless otherwise stated. Data were analyzed from October 2017 to January 2020.

## Results

One hundred fifty-six patients (112 [71.8%] men; mean [SD] age, 56 [12] years), including 81 in the ACP group and 75 in the sleep physician group, were enrolled between October 2014 and May 2017, all of whom were included in this analysis. Baseline demographic and clinical characteristics are presented in Table 1. The mean (SD) REI was 52 (28) events per hour, 123 patients (79%) were using continuous PAP therapy, and the mean (SD) ESS score was 10 (6).

**Table 1.** Baseline Characteristics of All Patients

Characteristic	Patients, No. (%)		
	All patients (N = 156)	Treated by physician (n = 75)	Treated by alternative care practitioner (n = 81)
Male	112 (71.8)	54 (72.0)	58 (71.6)
Age, mean (SD), y	56 (12)	55 (13)	54 (12)
Respiratory event index, mean (SD), events/h	52 (28)	55 (29)	51 (28)
Nocturnal oxygen saturation by pulse oximetry, mean (SD), %	85 (5)	86 (5)	85 (5)
Smoking status			
Active smoker	33 (21.2)	13 (17.3)	20 (24.7)
Former smoker	65 (41.7)	31 (41.3)	34 (42.0)
Nonsmoker	58 (37.2)	31 (41.3)	27 (33.3)
Pack-years of smoking, mean (SD)	28 (23)	26 (22)	31 (21)
Body mass index, mean (SD)^a^	39 (10)	39 (9)	40 (10)
Treatment type^b^			
Continuous positive airway pressure	123 (78.8)	59 (78.7)	64 (79.0)
Bilevel positive airway pressure	18 (11.5)	6 (8.0)	11 (13.6)
Oxygen	7 (4.5)	8 (10.7)	11 (13.6)
No therapy	8 (5.1)	6 (8.0)	4 (4.9)
Comorbidity			
Hypertension	96 (61.5)	47 (63.7)	49 (60.5)
Diabetes	42 (26.8)	21 (28.0)	21 (25.9)
Cardiovascular disease	31 (19.9)	16 (21.3)	15 (18.5)
Chronic lung disease	39 (25.0)	16 (21.3)	23 (28.4)
Chronic kidney disease	5 (3.2)	5 (6.7)	0
Epworth Sleepiness Scale score, mean (SD)	10 (6)	11 (6)	11 (5)
Sleep Apnea Quality of Life Index score, mean (SD)	5 (1)	4.6 (1.3)	4.5 (1.4)
Health Utilities Index Mark 2 score, mean (SD)	0.7 (0.2)	0.68 (0.3)	0.73 (0.18)
Health Utilities Index Mark 3 score, mean (SD)	0.6 (0.3)	0.56 (0.32)	0.59 (0.31)

^a^ Body mass index is calculated as weight in kilograms divided by height in meters squared.

^b^ Percentages for treatment type may sum to greater than 100% because some patients used oxygen in addition to continuous positive airway pressure or bilevel positive airway pressure.

The mean time from referral to initial visit was 88 days (95% CI, 79-96 days), and the mean time to treatment was 123 days (95% CI, 112-133 days) for all patients regardless of study group. Wait times from referral to initial assessment were statistically significantly shorter in the ACP group with a mean of 73 days (95% CI, 62-84 days) vs 102 days (95% CI, 90-114 days) (*P* < .001). Although the difference between study groups was diminished, the wait time from referral to treatment initiation remained shorter in the ACP group, with a mean of 110 days (95% CI, 94-125 days) vs 134 days (95% CI, 121-147 days) (*P* = .01).

Compared with nonadherent patients, patients who were adherent to treatment waited 15 fewer days (95% CI, 12-19 days) for initial assessment (*P* = .07) and 30 fewer days (95% CI, 23-35 days) for treatment initiation (*P* = .008) (Figure). Of patients whose treatment was initiated within 90 days, 72% were adherent to PAP therapy.

**Figure.**
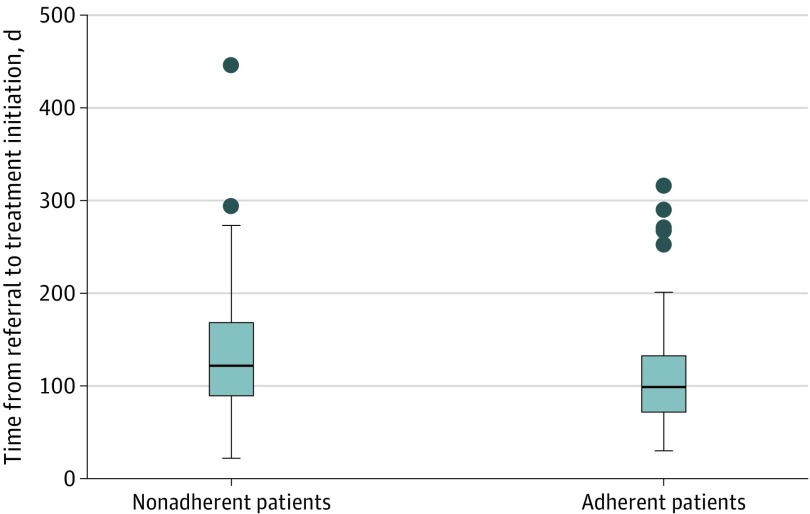
Time From Referral to Treatment Initiation, by Patient Adherence Box plot shows time to treatment initiation for adherent and nonadherent patients. Adherence was defined as positive airway pressure use of 4 or more hours on at least 70% of nights.^14^ The lines in the boxes denote medians, the tops and bottoms of the boxes denote the interquartile range, error bars denote adjacent values, and dots denote outliers.

### Factors Associated With Wait Times

Baseline characteristics that were associated with longer wait times for treatment included age, sex, number of comorbidities, number of medications, use of cardiovascular medications, study group, baseline REI, and baseline ESS score. Failure to attend an appointment for PSG was also associated with longer delays for treatment, but there was no association of missed clinician appointments with wait time for treatment initiation.

In the multivariable analysis (Table 2), male sex was associated with a 39-day delay in treatment initiation (mean coefficient, 38.64; 95% CI, 1.93 to 75.37; *P* = .04), and cardiovascular disease was associated with a 48-day delay (mean coefficient, 48.22; 95% CI, 3.13 to 93.31; *P* = .04). Similarly, missing an appointment for PSG was associated with a 48-day delay (mean coefficient, 47.93; 95% CI, 9.24 to 86.61; *P* = .02), and time to treatment initiation was 2.63 days shorter for every 1-point increase in ESS (mean coefficient, −2.63; 95% CI, −5.23 to −0.33; *P* = .05). Of note, study group was not associated with a reduced wait time to treatment after accounting for other covariates (mean coefficient, 13.37; 95% CI, −16.09 to 42.83; *P* = .37). These results were unchanged in a larger multivariable model that included body mass index, hypertension, diabetes, pulmonary disease, psychiatric comorbidity, individual medication classes (eg, sedatives or cardiovascular medications), baseline apnea-hypopnea index on PSG, and missed appointments with ACPs and sleep physicians and for PSG.

**Table 2.** Factors Associated With Wait Times for Treatment Initiation in Multivariable Analysis

Variable	Coefficient, mean (95% CI)	*P* value
Age	−0.42 (−1.96 to 1.11)	.59
Sex	38.64 (1.93 to 75.37)	.04
No. of comorbidities	−3.36 (−19.44 to 12.72)	.68
No. of medications	3.01 (−1.44 to 7.47)	.18
Baseline		
Apnea-hypopnea index score on polysomnogram	0.21 (−0.11 to 0.53)	.20
Epworth Sleepiness Scale	−2.63 (−5.23 to −0.33)	.05
Visit Specific Satisfaction Instrument–9 at 3 mo	−0.0039 (−0.0083 to 0.00057)	.09
Cardiovascular disease	48.22 (3.13 to 93.31)	.04
Study group	13.37 (−16.09 to 42.83)	.37
Missed appointment		
Physician	8.02 (−36.07 to 52.11)	.72
Alternative care practitioner	12.73 (−17.67 to 43.11)	.41
Appointment for polysomnogram	47.93 (9.24 to 86.61)	.02

### Factors Associated With PAP Adherence

Factors associated with adherence in the univariate analysis included smoking history, number of comorbidities, and wait time to treatment initiation. When controlling for covariates, only wait time to treatment initiation was associated with adherence to PAP therapy (odds ratio, 0.99; 95% CI, 0.98-0.99; *P* = .04) (Table 3). Results were unchanged in models that included additional comorbidity, medication, and attendance at appointments for clinicians or PSG. Per-protocol results were similar. Although the initial analysis included time to initial visit as a factor associated with adherence, this association was no longer statistically significant in the multivariable model once time to treatment initiation was included.

**Table 3.** Factors Associated With Adherence to Positive Airway Pressure Therapy at 3 Months in Multivariable Analysis^a^

Variable	OR (95% CI)	*P* value
Wait time to treatment	0.99 (0.98-0.99)	.04
Age	0.97 (0.92-1.02)	.32
Sex	1.72 (0.51-5.82)	.38
No. of comorbidities	0.66 (0.39-1.14)	.14
Cardiovascular disease	1.26 (0.26-6.14)	.78
No. of medications	1.14 (0.96-1.35)	.14
Neuropsychiatric medication	1.15 (0.51-2.58)	.74
Narcotic use	0.41 (0.12-1.41)	.16
Smoking history	0.77 (0.41-1.45)	.42
Study group	1.44 (0.52-3.93)	.48
Baseline		
Apnea-hypopnea index score on polysomnogram	1.01 (0.99-1.02)	.40
Respiratory event index during home sleep apnea testing	1.01 (0.99-1.03)	.31
Epworth Sleepiness Scale score	0.97 (0.89-1.06)	.56
Missed appointment for polysomnogram	0.38 (0.32-4.43)	.44

Abbreviation: OR, odds ratio.

^a^ Adherence was defined as positive airway pressure use for at least 4 hours on at least 70% of nights.

Notably, study group (ie, ACP clinic vs sleep physician) was not associated with adherence in either the univariate or multivariable models (odds ratio, 1.44; 95% CI, 0.52 to 3.93; *P* = .48) (Table 3). The interaction term for wait times and study arm was collinear with wait times measures and was not assessed further in the models. When assessed as a continuous variable (hours per night of PAP use), the multivariable models revealed an association between wait time and total days of PAP use (mean coefficient, −0.16; 95% CI, −0.31 to −0.01; *P* = .04); there was no association of wait times for treatment with nightly hours of use (mean coefficient, −0.01; 95% CI, −0.02 to 0.00; *P* = .06).

### Factors Associated With Patient-Reported Outcomes

Shorter wait time to treatment initiation was associated with greater improvement in patient-reported outcomes, including change in ESS score from baseline to 3 months (mean coefficient, −9.37; 95% CI, −18.51 to −0.24; *P* = .04) and Visit-Specific Satisfaction Instrument–9 score at 3 months (mean coefficient, −0.024; 95% CI, −0.047 to −0.0015; *P* = .04; (Table 4). These associations remained statistically significant despite the addition of treatment adherence to the models and did not change when additional demographic, comorbidity, or appointment attendance variables were added. Quality-of-life measures were not associated with wait times. Study group was not associated with patient-reported outcomes. Per-protocol results were similar.

**Table 4.** Factors Associated With Patient-Reported Outcomes at 3 Months in Multivariable Analysis

Variable	Change in Epworth Sleepiness Scale^a^		Visit-Specific Satisfaction Instrument–9^b^	
	Coefficient (95% CI)	*P* value	Coefficient (95% CI)	*P* value
Wait time to treatment	−9.37 (−18.51 to −0.24)	.04	−0.024 (−0.047 to −0.0015)	.04
Age	−40.27 (−92.63 to 12.08)	.13	−0.25 (−0.13 to 0.79)	.64
Sex	272.47 (−958.33 to 1503.27)	.66	0.73 (−2.03 to 3.48)	.60
No. of comorbidities	189.38 (−418.97 to 797.73)	.54	0.12 (−1.22 to 1.47)	.86
Cardiovascular disease	249.54 (−1481.01 to 1980.10)	.78	2.55 (−1.35 to 6.44)	.20
No. of medications	54.82 (−92.12 to 201.76)	.46	−0.17 (−0.45 to 0.11)	.24
Neuropsychiatric medication	252.59 (−783.69 to 1288.87)	.63	2.45 (0.056 to 4.85)	.05
Narcotic use	−1804.72 (−3402.73 to −206.71)	.03	−1.39 (−5.09 to 2.32)	.46
Study group	−428.36 (−1502.58 to 645.85)	.42	−1.65 (−4.13 to 0.82)	.19
Baseline				
Apnea-hypopnea index on polysomnogram	−0.000083 (−0.13 to 0.13)	.99	−0.0000019 (−0.00027 to 0.00026)	.99
Respiratory event index on home sleep apnea testing	11.69 (−10.13 to 33.52)	.29	−0.0046 (−0.050 to 0.041)	.84
Epworth Sleepiness Scale score	−75.38 (−174.87 to 24.12)	.14	−0.16 (−0.43 to 0.099)	.22
Adherence^c^	1843.26 (736.04 to 2950.47)	.001	4.10 (1.54 to 6.66)	.002
Change in Epworth Sleepiness Scale score at 3 mo	NA	NA	−0.29 (−0.56 to −0.018)	.04

Abbreviation: NA, not applicable.

^a^ Results are presented for patients with complete follow-up Epworth Sleepiness Scale data (127 total; 60 in the physician group and 67 in the alternative care practitioner group).

^b^ Results are presented for patients with complete Visit-Specific Satisfaction Instrument–9 data (106 total; 50 in the physician group and 56 in the alternative care practitioner group).

^c^ Adherence was defined as positive airway pressure use for at least 4 hours on at least 70% of nights.

## Discussion

The findings of this study suggest that adherence was higher in patients with severe SDB who had shorter wait times for treatment initiation. Furthermore, earlier initiation of therapy also led to greater benefits in sleepiness and visit-specific satisfaction. Although certain patient factors, such as male sex and comorbid chronic disease, were shown to be associated with wait times, there remained an association between wait times for treatment and improved clinical outcomes. To our knowledge, this is the first study to examine the association of wait times for SDB treatment with clinical outcomes, demonstrating that for patients with severe SDB, earlier initiation of treatment improves treatment adherence and patient-reported outcomes.

These results are consistent with prior literature in other diseases, which suggests an association between long delays for health care and adverse clinical outcomes.^16^ Sobolev et al^17^ examined the effect of wait times on patients for coronary artery bypass graft surgery and found an increased risk of early death for all patients, including those with less severe disease. In a follow-up study,^18,19^ the same authors assessed the impact of prioritizing patients awaiting coronary artery bypass graft on the basis of clinical urgency and reported that longer delays contributed to higher all-cause mortality before surgery.

Other studies have assessed additional negative consequences of long wait times, such as increased morbidity, workplace attendance, decreased quality of life, more frequent hospitalizations, and higher health care costs. Long wait times for knee replacement have been linked to greater pain, functional limitation, and decreased quality of life.^20^ Prolonged wait times for cataract surgery have been associated with further vision loss, greater risk of falls, and the potential for decreased efficacy after treatment.^21^ Waaijer et al^22^ demonstrated the adverse effect of wait times on tumor size, clinical stage, and response to radiotherapy in patients with oropharyngeal carcinoma. Reductions in general health, long-term addiction to painkillers and narcotics, and increased risk of depression while waiting for care have also been reported.^16^ We did not directly explore the association of wait times with long-term clinical consequences of untreated SDB; however, the role of PAP therapy in mitigating downstream risk highlights the importance of improving adherence through timely initiation of treatment.

In previous studies,^23,24^ long wait times worsened patient outcomes by delaying definitive medical or surgical interventions that were not dependent on patient behaviors. The current study is unique in that it identified a direct association between delays for treatment and adherence to therapy, which is an important behavioral aspect of chronic disease management. These findings suggest that innovative models of care delivery to improve timely access may influence patient behavior, leading to improved adherence to therapy and better clinical outcomes.^23,24^ Such interventions could be implemented alone or along with other targeted behavioral strategies to improve treatment adherence,

There are several possible explanations for the association of earlier treatment initiation with PAP adherence. First, shorter delays from referral to treatment initiation may have highlighted the importance of PAP treatment for patients, thereby increasing motivation to use PAP compared with those who experienced longer delays for care. A similar phenomenon has been demonstrated for patients with psychiatric illness, who were at higher risk of missing a scheduled appointment with increasing delay from the time of referral.^25^ Shorter wait times may have improved the patient experience, leading to greater patient engagement in their care, as has been demonstrated in other chronic diseases.^26^ Although patient experience was not measured directly, the positive association between wait time to treatment initiation and patient-reported outcomes, including patient satisfaction, suggests that the experience may also have been favorable. Conversely, patients may have interpreted long delays for care as indicative of relative unimportance of SDB, leading to poorer adherence to therapy. Future studies exploring patient experience with care could aim to capture patient perspectives on wait times and the importance of SDB treatment.

It is also possible that patients who were likely to be adherent to PAP were also more motivated to expedite treatment initiation. Although we were unable to measure these motivational factors directly, we explored attendance at appointments as a surrogate of this shared adherence effect. Although missed appointments for PSG were associated with an increased wait time to treatment initiation, there was no association between missed clinician or PSG appointments and adherence or patient-reported outcomes. The results of this analysis, which included appointment attendance, suggest a contribution of wait times that is independent of a possible shared motivation for all aspects of care.

We also found that a shorter wait time for treatment initiation was associated with greater improvements in ESS. This finding may reflect improved patient experience from more timely care, or may be associated with PAP adherence, as has been demonstrated previously in observational analyses that did not include wait time as a covariate.^27^ In the current analysis, the persistence of a statistically significant relationship when PAP adherence was included in the models suggests an independent contribution of wait times to symptom improvement. Overall, it is likely that shorter wait times moderate symptomatic improvement both independently and through greater PAP adherence.

Care in the ACP clinic was associated with shorter wait times for initial assessment and for treatment initiation compared with care by sleep physicians. Thus, it is possible that the association of shorter wait time with symptom improvement was driven by factors related to the ACP model of care, such as increased contact with ACPs or better experience with ACP care. Supporting this assertion is the finding that improvements in ESS were greater in the ACP group. However, the association of ACP care with wait time was diminished between initial visit and treatment initiation, and the association of study group with time to treatment was negated when covariates such as male sex, excessive sleepiness, cardiac disease, and delayed PSG were included. This finding suggests that an earlier initial visit with an ACP did not necessarily result in earlier time to treatment initiation once patient-related and system-related factors were considered. Overall, improvements in sleepiness are unlikely to have been associated with the model of care alone.

### Limitations

This study has several limitations. First, this was a study conducted in a single, academic sleep center with an established ACP program for patients with SDB. Furthermore, patients had severe SDB; thus, the results should be interpreted in this context and may not be generalizable to other patient populations or clinical settings. However, the notion that wait times may be associated with downstream treatment adherence in patients with chronic disease warrants further exploration. Second, even within the context of an RCT aiming to improve access, patients with severe SDB still waited longer than the recommended 4 weeks to be assessed. It is possible that the magnitude of the beneficial associations of earlier contact may be underestimated when the wait times achieved were still outside of what is considered acceptable by sleep specialists and, likely, by the public as well. Furthermore, as a secondary analysis of a randomized clinical trial, this study may have been underpowered to detect associations of wait times with more clinically important measures of adherence, such as nightly hours of PAP use; similarly, we were unable to establish percentile thresholds for PAP adherence that might be useful for quality measurement and benchmarking. Prospective, adequately powered studies could be undertaken in the future to increase confidence in these findings.

## Conclusions

To our knowledge, this study is the first to identify that reducing wait times independently affects treatment adherence, symptoms, and patient satisfaction in patients with SDB. These findings suggest that system interventions could be used to modify patient behavior, leading to improved adherence and better clinical outcomes. Strategies to improve wait times could improve patient experience, clinical outcomes, and subsequently reduce costs. Such innovations would align strongly with the triple aim (ie, improving the patient experience of care, improving the health of populations, and reducing the per-capita cost of health care) for optimizing health care quality.

## Notes

Supplement 1. **Trial Protocol**

Supplement 2. **eFigure.** Overview of Study Design

Supplement 3. **Data Sharing Statement**

## References

[1] Deak MC, Kirsch DB Sleep-disordered breathing in neurologic conditions. Clin Chest Med. 2014;35(3):-. doi:10.1016/j.ccm.2014.06.009 25156770

[2] Heinzer R, Vat S, Marques-Vidal P, Prevalence of sleep-disordered breathing in the general population: the HypnoLaus study. Lancet Respir Med. 2015;3(4):310-318. doi:10.1016/S2213-2600(15)00043-0 25682233 PMC4404207

[3] Peppard PE, Young T, Barnet JH, Palta M, Hagen EW, Hla KM Increased prevalence of sleep-disordered breathing in adults. Am J Epidemiol. 2013;177(9):1006-1014. doi:10.1093/aje/kws342 23589584 PMC3639722

[4] Marshall NS, Wong KK, Liu PY, Cullen SR, Knuiman MW, Grunstein RR Sleep apnea as an independent risk factor for all-cause mortality: the Busselton Health Study. Sleep. 2008;31(8):1079-1085. 18714779 PMC2542953

[5] OECD Health at a glance 2017: OECD indicators. Published November 10, 2017 Accessed March 16, 2020. https://www.oecd.org/social/health-at-a-glance-19991312.htm

[6] Tseng VL, Yu F, Lum F, Coleman AL Risk of fractures following cataract surgery in Medicare beneficiaries. JAMA. 2012;308(5):493-501. doi:10.1001/jama.2012.9014 22851116

[7] Statistics Canada Statistics Canada: access to health care services in Canada. Published 2003 Accessed September 3, 2019. https://www150.statcan.gc.ca/n1/en/pub/82-624-x/2016001/article/14683-eng.pdf?st=s2RSk2iD

[8] Flemons WW, Douglas NJ, Kuna ST, Rodenstein DO, Wheatley J Access to diagnosis and treatment of patients with suspected sleep apnea. Am J Respir Crit Care Med. 2004;169(6):668-672. doi:10.1164/rccm.200308-1124PP 15003950

[9] Pendharkar SR, Tsai WH, Penz ED, A randomized controlled trial of an alternative care provider clinic for severe sleep-disordered breathing. Ann Am Thorac Soc. 2019;16(12):1558-1566. doi:10.1513/AnnalsATS.201901-087OC 31437008

[10] Povitz M, Kendzerska T, Hanly PJ, Profile of CPAP treated patients in Ontario, Canada, 2006-2013: a population-based cohort study. Sleep Med. 2018;51:22-28. doi:10.1016/j.sleep.2018.06.005 30081383

[11] Rotenberg B, George C, Sullivan K, Wong E Wait times for sleep apnea care in Ontario: a multidisciplinary assessment. Can Respir J. 2010;17(4):170-174. doi:10.1155/2010/420275 20808975 PMC2933773

[12] Fleetham J, Ayas N, Bradley D, ; Canadian Thoracic Society Sleep Disordered Breathing Committee Canadian Thoracic Society 2011 guideline update: diagnosis and treatment of sleep disordered breathing. Can Respir J. 2011;18(1):25-47. 21369547 10.1155/2011/506189 PMC3070752

[13] Ip-Buting A, Kelly J, Santana MJ, Evaluation of an alternative care provider clinic for severe sleep-disordered breathing: a study protocol for a randomised controlled trial. BMJ Open. 2017;7(3):e014012. doi:10.1136/bmjopen-2016-014012 28360244 PMC5372098

[14] Kribbs NB, Pack AI, Kline LR, Objective measurement of patterns of nasal CPAP use by patients with obstructive sleep apnea. Am Rev Respir Dis. 1993;147(4):887-895. doi:10.1164/ajrccm/147.4.887 8466125

[15] Ware JE Jr, Hays RD Methods for measuring patient satisfaction with specific medical encounters. Med Care. 1988;26(4):393-402. doi:10.1097/00005650-198804000-00008 3352332

[16] Barua B, Esmail N, Jackson T The Effect of Wait Times on Mortality in Canada. Fraser Institute; 2014.

[17] Sobolev BG, Levy AR, Kuramoto L, Hayden R, Brophy JM, FitzGerald JM The risk of death associated with delayed coronary artery bypass surgery. BMC Health Serv Res. 2006;6:85. doi:10.1186/1472-6963-6-85 16822309 PMC1574305

[18] Sobolev BG, Kuramoto L, Levy AR, Hayden R Cumulative incidence for wait-list death in relation to length of queue for coronary-artery bypass grafting: a cohort study. J Cardiothorac Surg. 2006;1:21. doi:10.1186/1749-8090-1-21 16930475 PMC1564012

[19] Sobolev BG, Levy AR, Kuramoto L, Hayden R, FitzGerald JM Do longer delays for coronary artery bypass surgery contribute to preoperative mortality in less urgent patients? Med Care. 2006;44(7):680-686. doi:10.1097/01.mlr.0000220257.81482.67 16799363

[20] Desmeules F, Dionne CE, Belzile EL, Bourbonnais R, Frémont P The impacts of pre-surgery wait for total knee replacement on pain, function and health-related quality of life six months after surgery. J Eval Clin Pract. 2012;18(1):111-120. doi:10.1111/j.1365-2753.2010.01541.x 21040250

[21] Conner-Spady B, Sanmartin C, Sanmugasunderam S, A systematic literature review of the evidence on benchmarks for cataract surgery waiting time. Can J Ophthalmol. 2007;42(4):543-551. doi:10.3129/i07-094 17641695

[22] Waaijer A, Terhaard CH, Dehnad H, Waiting times for radiotherapy: consequences of volume increase for the TCP in oropharyngeal carcinoma. Radiother Oncol. 2003;66(3):271-276. doi:10.1016/S0167-8140(03)00036-7 12742266

[23] Stepnowsky CJ Jr, Marler MR, Ancoli-Israel S Determinants of nasal CPAP compliance. Sleep Med. 2002;3(3):239-247. doi:10.1016/S1389-9457(01)00162-9 14592213

[24] Stepnowsky CJ, Marler MR, Palau J, Annette Brooks J Social-cognitive correlates of CPAP adherence in experienced users. Sleep Med. 2006;7(4):350-356. doi:10.1016/j.sleep.2005.11.004 16713349

[25] Gallucci G, Swartz W, Hackerman F Impact of the wait for an initial appointment on the rate of kept appointments at a mental health center. Psychiatr Serv. 2005;56(3):344-346. doi:10.1176/appi.ps.56.3.344 15746510

[26] Robinson JH, Callister LC, Berry JA, Dearing KA Patient-centered care and adherence: definitions and applications to improve outcomes. J Am Acad Nurse Pract. 2008;20(12):600-607. doi:10.1111/j.1745-7599.2008.00360.x 19120591

[27] Pendharkar SR, Dechant A, Bischak DP, Tsai WH, Stevenson AM, Hanly PJ An observational study of the effectiveness of alternative care providers in the management of obstructive sleep apnea. J Sleep Res. 2016;25(2):234-240. doi:10.1111/jsr.12358 26503454

