# Supplementary material for: Effects of Wait Times on Treatment Adherence and Clinical Outcomes in Patients With Severe Sleep-Disordered Breathing: A Secondary Analysis of a Noninferiority Randomized Clinical Trial
Source: JAMA Netw Open. 2020 Apr 20;3(4):e203088. doi: 10.1001/jamanetworkopen.2020.3088 (PMC7171552; doi:10.1001/jamanetworkopen.2020.3088)
Supplement: Supplement 1. — Trial Protocol [file jamanetwopen-3-e203088-s001.pdf]

FAST TRACK PROTOCOL

RECRUITING PROCESS

TRIAGING PROCESS

Triage team will screen all referrals to identify all suspected sleep disordered breathing (SDB) patients with one of the following criteria:

- "Urgent" categorized referral. If patient is deemed urgent based on ESS alone, the Fast Track clinic supervisor will review with the project's lead sleep physician (LSP) to determine whether assessment for study eligibility is appropriate
- No previous treatment of sleep apnea (i.e. CPAP, BPAP, dental appliance, upper airway surgery)
- Adjusted neck circumference (ANC) >= 43 from the referral questionnaire. ANC = Neck circumference in cm + 3 cm (history of snoring) + 3 cm (history of witnessed apnea) + 4 cm (Hypertension)
- A level 3 home sleep apnea test (HSAT) result with one of the following:
  - Respiratory disturbance index (RDI) > 30 events/hour
  - Mean nocturnal oxygen saturation < 85%
  - Suspected hypoventilation, defined by an RDI > 15 events/hour AND partial pressure of carbon dioxide > 45 mmHg on arterial blood gas (within 6 months prior to referral).

The triaging team will give the triaged referrals to the booking clerk to schedule ambulatory sleep tests for each patient. All triaged HSAT appointments will be marked in green in the scheduling system. The research associate (RA) will check the scheduling system (daily) and/or with the booking clerk to obtain date and times of HSAT appointments. **The RA cannot see referrals or any patient information at this point until the sleep centre's general research consent form is signed**.

**Possible scenarios**	**Action**
Patient has one or more concerns that may compromise enrollment (i.e. Patients with significant cognitive impairment)	The triaging team will notify the LSP to review the patient's chart and determine if patient should be approached for the study.<br><br>The triaging team will notify the RA if the patient is deemed ineligible on this basis and the RA will manually tally the number and reasons for ineligibility.
Patients referred with unexplained hypoxemia who are on oxygen	The triaging team will notify the LSP to determine if patient meets triaging criteria. (e.g. suspected severe SDB vs. other cardiopulmonary disease requiring oxygen and a suspicion of sleep disorder)<br><br>The LSP will also determine if the patient requires an additional

	HSAT without oxygen. If so, the LSP will provide a note in the chart and the booking clerk will book the patient.
Out of town patients	The triaging team will accept out-of-house HSAT tests provided that the tests are completed with a Remmers recorder. The LSP will review the patient's chart and test to authorize the inclusion of these patients/tests before the patients are contacted.

Consenting Process

Upon arrival to the ambulatory sleep test appointment, the patient will be given the opportunity to sign the general research consent form by the HSAT administration team. Once patient has completed the HSAT training process and has signed the green form, HSAT clerk will say the following to the patient:

> "Based on your profile, you may be eligible to participate in a study. [Name] is a research associate and he/she is available in the waiting area to tell you what this study is about. Your participation is appreciated but not mandatory."

If the patient agrees, the RA will greet the patient and explain and obtain a written informed consent for the Fast Track study.

Upon consenting, the RA will prepare a hard-copy of the "Fast Track Summary Sheet" (see Appendix 1). All data will also be entered into an Excel sheet. All hard-copies of the "Fast Track Summary Sheet" and the Excel sheet will be stored at W21C.

**Possible scenarios**	**Action**
Patient does not sign the general research consent form	The RA will have no access to patient chart/data and will manually tally the number of non-consenting individuals manually. The RA will notify the Fast Track clinic supervisor of patient (anonymously).<br><br>The LSP will review all charts with the Fast Track clinic supervisor to determine if the assessment will be done with the ACP Fast Track clinic (non-study) or by a sleep physician.
Patient consent to general research but not to the study.	The RA will ask why patient does not want to participate in the study and will tally the number and reason of non-consenting patients. The RA will notify the Fast Track clinic supervisor of patient.<br><br>The LSP will review all charts with the Fast Track clinic supervisor to determine if the assessment will be done with the ACP Fast Track clinic (non-study) or by a sleep physician.
Patient cannot stay after HSAT training to complete the consenting process	The RA will provide the following options to the patient:<br>1) Explain and obtain a verbal consent over the phone, followed by a written consent at the baseline visit (regardless of eligibility),<br>2) Explain and obtain written consent when patient returns

	the HSAT machine,<br>3) Give the consent form to the patient to read/sign at home and return when patient returns the machine.<br><br>If patient refuses all options, patient is opted out of the study. The RA will notify the Fast Track clinic supervisor of patient and tally the number and reason of non-consenting patients.<br><br>The LSP will review all charts with the Fast Track clinic supervisor to determine if the assessment will be done with the ACP Fast Track clinic (non-study) or by a sleep physician.
Patient expresses difficulty in availability	The RA will let patients know that the questionnaire portion of the study could be mailed/emailed to the patient and HSAT could be completed via homecare company.<br><br>If patient still expresses difficulty, the RA will check if the patient would rather opt out of the study.
Patient requires more time to understand and read through the consent form.	The patient will be given a consent form to take home and return after completing the ambulatory sleep test. If a consent form is not returned within 24 hours, the RA will call to follow-up with the patient.
Patients who are eligible based on previous HSAT results	The Fast Track clinic supervisor will contact the patients via phone to obtain verbal consent for general research. If patient provides verbal consent, the Fast Track clinic supervisor will introduce the study and inform that an RA will be contact the patient. The RA will call the patient for a verbal consent for the study. If patient provides a verbal consent, a written consent will be obtained during the patient's baseline visit regardless of eligibility.

Note: Unlike standard referral procedures, the ambulatory sleep test and provider appointment will not be scheduled simultaneously, since the assignment of provider and subsequent appointment availability will depend on the study arm to which the patient is randomized.

ELIGIBILITY

Eligibility criteria include:

- Written consent signed by the patient,
- Patient is over 18 years old,
- Patient did not have previous treatment of SDB,
- HSAT results must show one of the following:
  - RDI > 30 events/hour on an ambulatory sleep test
  - Mean nocturnal oxygen saturation < 85%
  - Suspected hypoventilation, defined by an RDI > 15 events/hour <u>AND</u> partial pressure of carbon dioxide > 45 mmHg on arterial blood gas.

Exclusion criteria include:

- Primary health insurance provided by a province other than Alberta
- No previous treatment of sleep apnea (i.e. CPAP, BAP, dental appliance, upper airway surgery)

The RA will make a copy of HSAT results to include with the "Fast Track Summary Sheet" for each consented patient. The RA will notify The LSP to review HSAT results for **all patients regardless of eligibility** within a week. If patient is eligible, the RA will notify patients of eligibility and to expect a call from the sleep centre clerks within 5 – 10 business days.

If patient is not eligible, the RA will give the patient's chart to The Fast Track clinic supervisor. The LSP will review all charts with The Fast Track clinic supervisor to determine if the assessment will be done with the ACP Fast Track clinic (non-study) or by a sleep physician.

**Possible scenarios**	**Action**
Patient has multiple HSAT results	The most recent HSAT results from the clinic's scheduled HSAT will be used to determine eligibility.
Patient no longer wants to participate in the study or is not eligible	The RA will attempt to identify the reason why patient does not want to participate in the study. The RA will remove the patient from the study and manually tally the number and reason the patient exited the study.<br><br>Patient without a baseline visit will not be counted as part of the study. The RA will notify the Fast Track clinic supervisor of patient.
Patient provided verbal consent but is not eligible	Patient will still need to provide a written consent. The RA will approach the patient when patient is booked to review HSAT results.

RANDOMIZATION

Eligible patients will be randomly assigned by the RA to either to the Fast Track arm (intervention group) or to the standard treatment arm (control group) using the randomization website (www.randomization.com).

For patients assigned to the "Fast Track" arm, the Fast Track clinic supervisor will schedule an appointment for the patient with an ACP. For patients assigned to the standard treatment arm, the booking clerk will schedule an appointment for the patient with a sleep physician. Patients will be asked to arrive 15 - 20 minutes prior to the appointment to complete study questionnaires. The booking clerks will notify the RA of the initial appointment date and time.

STUDY PROCEDURE

BASELINE VISIT & TREATMENT PLAN

When patient arrives, the RA will ask the patients to complete the following questionnaires (ESS, Short SAQLI without treatment section, and HUI). The study will require an ESS at the time of the baseline visit, even though there may be multiple ESS questionnaires in the patient's chart.

If required, the sleep physician will order a polysomnography (PSG). The RA will check that the PSG is ordered as "urgent". The following will be collected: PSG date, AHI, mean oxygen saturation and percentage of time under 90%.

The RA will access the CPAP database to retrieve the patient's treatment type. The RA will retrieve the patient's treatment start date for the following treatments by:

- CPAP/BAP – retrieving and calling the homecare company OR CPAP database,
- Oxygen only – looking at the patient's chart for the date when the patient was contacted about the PSG results (if the patient is previously on oxygen) OR determining when the patient started on oxygen,
- Dental appliance – calling the dentist,
- Weight loss – looking in the patient's chart for the date treatment options were offered

Possible scenarios	Action
Patient cancels or does not show to the appointment	The RA will note the original appointment date and the rescheduled date.
Patient does not show for 3 or more appointments	Patient is discharged from the CPAP clinic and the study.
Patient stops their initial treatment	The RA will note new treatment. 3-month and 1-year appointments will still be based on the original treatment date.
Multiple patients need to meet the RA at the same time (recruitment, clinical visits)	Possible options:<br>1) Ask an another RA to assist in one of the appointments<br>2) Ask The Fast Track clinic supervisor to obtain clinical study data at visit
Patients with only a verbal consent to the study	The RA will review the study with the patient and obtain a written consent to the patient regardless if the patient is eligible or not.<br><br>If the patient revokes their verbal consent, the RA will remove patient from the study, identify the reason why the patient does not want to participate in the study. Because the patient has not yet had an initial appointment with a physician or an ACP, the patient will not be counted as part of the 150 patients recruited.<br><br>The RA will notify the Fast Track clinic supervisor of patient.
Patient delays their treatment	If the patient delays their treatment due to insurance purposes, treatment start date will be the date they start their treatment.<br><br>If the patient delays their treatment due to other medical reasons, the treatment start date will be the date that treatment was offered. Three-month and one year follow-up data will be collected as per usual procedures.
Patient has had multiple	The RA will pull the file and review with the LSP as the treatment

PSGs after the indicated treatment start date	start date may need to be modified.
Patient does not have sleep disordered breathing after further testing (e.g., PSG)	The treatment start date will be the date of the diagnostic test. Three-month and one year follow-up data will be collected as per usual procedures.

3-Month & 1-Year Follow-Up (± 2 weeks)

3-Month Appointment Date and Time
The RA will create a list (weekly) of patients who have completed their initial appointment for the booking clerk. The booking clerk will review the scheduling system to book and/or confirm patient's 3-month appointment. Upon receiving the appointment date, the RA will complete a HSAT requisition for the booking clerk to book patient for a HSAT (in-house or with the patient's CPAP company – if ordering a HSAT from the home care provider, state on cover letter to send the raw data to FMC Sleep Centre) on the day of the follow-up appointment or whenever is most convenient for the patient. HSAT test is to be completed with a Remmers recorder. Note: if the patient is on O2, request the HSAT done on room air or without O2. If the HSAT is completed on O2 note make a note that it was completed on O2 on the patients chart.

1-Year Appointment Date and Time
The RA will create a list (every 3 months) of patients who have completed their 3-month follow-up appointment for the booking clerk. The booking clerk will create a tracking task in the scheduling system to book the appointment several months before the 1-year target date. When the appointment is book, booking clerk will notify RA of appointment date and time. Upon receiving the appointment date, RA will complete a HSAT requisition for the booking clerk to book patient for a HSAT (in-house or with the patient's CPAP company) on the day of the follow-up appointment or whenever is most convenient for the patient. HSAT test is to be completed with a Remmers recorder.

The RA will attempt to call all three month and one year patients who do not have a follow up appointment booked through the Sleep Centre to coordinate the best date and time to complete the questionnaires.

If the patient does not have a follow-up appointment booked at the Sleep Centre. We will use the date that the patient returns their questionnaire as the final appointment date; however, the research associate should note that it is the follow-up date not the final appointment date.

At Time of Appointment
The RA will give the patient a questionnaire package (ESS, Short SAQLI including treatment section, HUI, VSQ-9) at the time of visit. The ESS, Short SAQLI including treatment section and the HUI surveys are to be completed before the patient enters the clinical appointment. The VSQ9 survey is to be completed after the patient completes their clinical appointment. Download data will be

obtained by the Fast Track clinic supervisor or by the therapist. The patient will not be compensated for parking if the follow-up visit is part of the patient's clinical care.

If there are any information missing from the Fast Track Summary sheet (i.e. Triaging criteria, co-morbidities or medication), the RA will retrieve them from the chart or from the CPAP database before/during the time of visit.

**Possible scenarios**	**Action**
Patient has a follow-up appointment booked outside of the 3-month or 1-year period	The RA will book a follow-up appointment outside of the CPAP clinic with the patient and provide a reminder phone call a week before the appointment. The RA will also book a HSAT as per normal procedure.<br><br>At the appointment, the RA will:<br>1. Ask patient to complete the questionnaire package<br>2. Ask The Fast Track clinic supervisor or the home-care company to retrieve CPAP data from the patient's machine<br>3. Give the patient a pre-paid parking voucher.
Patient cannot come in for a follow-up appointment.	The RA will book a HSAT via home-care company.<br><br>The RA will email, fax, OR book a phone appointment with the patient to complete the questionnaire package. The date that the patient returns their questionnaire will be considered the final appointment date (3-month and 1-year); however, the RA should note that it is the follow-up date not the final appointment date.<br><br>The RA will phone the home-care company to retrieve CPAP data from the patient's machine.
HSAT cannot be booked on the same day as the 3 month or 1 year follow-up appointment.	The booking clerk will book a HSAT closest date to the follow-up appointment. The RA will give the patient a pre-paid parking voucher if the test was not ordered as part of the patient's clinical care.
Patient randomized in the "Fast Track" treatment arm requests to be seen by a physician.	The RA will confirm that participant still wishes to participate in the study. If so, then the patient will remain in the assigned study arm (as per the modified intention to treat design), and physician visits will be tracked as per study outcomes.<br><br>If the participant requests to withdraw from the study and withdraws from having their health utilization data collected, then no further research data will be collected. The patient will be informed that we will not collect questionnaires, additional clinical and HC utilization/cost data. Data up to the point of withdrawal is accessible to the research team. Everything else will be the same in terms of clinical care (i.e. dropping out of the Fast Track arm doesn't necessarily mean that all subsequent visits are with a physician, but rather that the patient goes back into standard care – physician-led but with heavy ACP involvement)
Patient is without a three-month or 12-	The RA will either call patient to come in for a follow-up to complete the follow-up data collection. If participant cannot meet with the RA,

month clinical follow-up, or has follow-up appointments that are not at 3 or 12 months	a questionnaire package will be mailed for the participant to complete. The date that the patient returns their questionnaire as the final appointment date; however, the research associate should note that it is the follow-up date not the final appointment date; however, the RA should note that it is the follow-up date not the final appointment date. Download data will be obtained through the participants PAP provider.<br><br>The RA will give the patient a pre-paid parking voucher for all appointments that are outside of standard clinical visits
Patient is discharged by the clinic	The RA will approach the patient to continue the study.
Patient withdraws from the study after being discharged by the ACP/Provider	The RA will ask permission to collect their data on healthcare utilization and visits to the sleep centre.<br><br>If participant declines, the RA will cease ongoing data collection. Data collected up to the point of participant withdrawal will be retained.
Cannot contact patient (i.e. called once a week for a month)	The RA will send a participation letter to the patient requesting that they contact the RA to coordinate a time to complete the questionnaires and HSATs. If the RA does not hear from the patient, they will be considered lost to follow-up.
Patient does not bring their PAP machine to the appointment	The patient will be called to bring the machine in to be downloaded by a therapist. If they are unable to make it in to the FMC Sleep Centre, a download compliance form will be faxed to the provider requesting the needed information be sent to the FMC Sleep Centre addressed to the booking clerk. The RA will provide the booking clerk with a list of all requested downloads and HSATs which she will flag and provide to the RA.

DATA COLLECTION

SUMMARY SHEET

	Data to collect	Data source	Photocopy, handwritten or original copy
Recruitment	Study consent	From patient during HSAT appointment	Original copy
	Triage Criteria (Level 3 HSAT or ANC)	Level 3 HSAT – patient's chart, if any.<br>ANC – Patient Questionnaire.	Handwritten
	HSAT – RDI, Mean O2 saturation, mean O2 saturation, time under 90%	HSAT Summary page, day after patient completes HSAT.	Photocopy
	Partial pressure of CO2 on ABG	Netcare – will need to ask a person with access to Netcare (The Fast Track clinic supervisor or Carly)	Photocopy
	HSAT Date	Chart	Handwritten
Baseline Visit	Initial baseline visit date	Sleep clinic's CIS system.	Handwritten
	Actual baseline visit date		
	ESS	At baseline visit.	Original
	Health Utilities Index		
	Short SAQLI (without treatment section)		
	Current medications	Patient's chart: 1) Patient info sheet (yellow) when patient comes in for the HSAT, 2) Physician's initial visit letter 3) Patient's self-filled questionnaire, 4) Administrative data from AHS	Handwritten
	Comorbidities		
	PSG date (if applicable)	Sleep clinic's CIS system.	Handwritten
	PSG - AHI, mean O2 saturation, time under 90%	Patient's chart - PSG report	Handwritten
	Treatment start date	CPAP database, homecare company (CPAP/BAP), patient's chart (Oxygen), dentist (dental appliance)	Handwritten
	Treatment type	CPAP database	Handwritten

	Data to collect	Data source	Photocopy, handwritten or original copy
3-Month/1-Year Follow-up	Initial 3-month/1-year visit date	Sleep clinic's CIS system.	Handwritten
	Actual 3-month/1-year visit date		
	ESS	At 3-month and 1-year visit.	Original copy
	Health Utilities Index		
	Short SAQLI (with treatment section)		
	Positive airway pressure adherence (1 month directly prior to the appointment) & date	At 3-month and 1-year visit OR via home-care company	Photocopy
1-Year only	Healthcare utilization - cumulative sum of outpatient physician visits, hospitalizations and length of stay, and urgent care/emergency room visits	DIMR Database	Electronic copy
	Healthcare utilization (part of a separate quality improvement initiative) – additional physician consultation time	Patient's chart: Written communication between ACPs and physicians are recorded on a yellow sheet.	Only tallies from the original copy
	Healthcare costs – costs for sleep physician and ACP visit, diagnostic sleep testing costs for HSAT and PSG	Taken from the Alberta Health Schedule of Medical Benefits, Health Sciences Association of Alberta (HSAA) Collective Agreement and the FMC sleep Sleep Centre Medical Director	Electronic copy
	Healthcare costs – CPAP therapy costs	Current market pricing through respiratory homecare providers	Electronic copy
	Healthcare costs – hospitalization costs	DIMR and AHS Finance department	Electronic copy
	Healthcare costs – costs for ambulatory visits	Canadian Institute for Health Information (CIHI) and DIMR	Electronic copy

*__Note: All handwritten, photocopied and original information will be transcribed to electronic format by RA and all data will be stored at the W21C.__

APPENDIX 1

"Fast Track" Summary Sheet

Study consent: ☐ Yes
☐ No

Triage Criteria: ☐ Level 3 HSAT
☐ ANC = NC______, HBP__, HSnore __, HApnea___

Referral Date: ______________________

Eligibility process

☐ Patient >= 18 years old
☐ Patient does not have a previous treatment of SDB

One of the following:

☐ RDI >= 30 events/hr
☐ Mean nocturnal O2 saturation <= 85%
☐ RDI >= 15 events/hr AND partial pressure of CO2 >=45 mmHg on ABG

Eligible: ☐ Yes: ☐ACP ☐Resp
☐ No

Baseline visit date: ______________________

☐ ESS
☐ Health Utilities Index
☐ SAQLI
☐ Current medications (if any)
☐ Comorbidities

PSG: ☐ Yes, date____________________
☐ No

Current Medications (list):

Comorbidities (list):

☐ High Blood Pressure	☐ Heart Failure
☐ Asthma	☐ Other Lung Disease
☐ Diabetes	☐ Dialysis
☐ Depression	☐ Fibromyalgia
☐ Angia/Heart Attack	☐ Heart Disease
☐ COPD/Emphysema	☐ Stroke
☐ Kidney Disease	☐ Atrial Fibrillation/ Arrhythmia
☐ Psychiatric Disease	

ID#: ____________________

Treatment date: ______________________

Initial 3mth Appointment Date: _____________________

Actual 3mth Appointment Date: _____________________
Checklist:

☐ ESS
☐ Health Utilities Index
☐ SAQLI
☐ Positive airway pressure adherence **(1 month)**
☐ Ambulatory sleep test
☐ CAHPS-GC

Questionnaire Mailing Dates:

☐ Yes. Mail date____________________
Receive date________________
☐ No

Initial 1 yr Appointment Date: _____________________

Actual 1 yr Appointment Date: _____________________
Checklist:

☐ ESS
☐ Health Utilities Index
☐ SAQLI
☐ Positive airway pressure adherence **(1 month)**
☐ Ambulatory sleep test
☐ CAHPS-GC
☐ Healthcare utilization

Questionnaire Mailing Dates:

☐ Yes. Mail date____________________
Receive date________________
☐ No
