# Supplementary material for: Effects of Wait Times on Treatment Adherence and Clinical Outcomes in Patients With Severe Sleep-Disordered Breathing: A Secondary Analysis of a Noninferiority Randomized Clinical Trial
Source: JAMA Netw Open. 2020 Apr 20;3(4):e203088. doi: 10.1001/jamanetworkopen.2020.3088 (PMC7171552; doi:10.1001/jamanetworkopen.2020.3088)
Supplement: Supplement 2. — eFigure. Overview of Study Design [file jamanetwopen-3-e203088-s002.pdf]

Supplementary Online Content

Thornton CS, Tsai WH, Santana MJ, et al. Effects of wait times on treatment adherence and clinical outcomes in patients with severe sleep-disordered breathing: a secondary analysis of a noninferiority randomized clinical trial. *JAMA Netw Open*. 2020;3(4):e203088. doi:10.1001/jamanetworkopen.2020.3088

eFigure. Overview of Study Design

This supplementary material has been provided by the authors to give readers additional information about their work.

© 2020 Thornton CS et al. *JAMA Network Open.*

eFigure. Overview of Study Design. Figure modified from Ip-Buting, et al (12). Total of 156 patients with 75 in the sleep physician arm and 81 in the alternative care provider (ACP) arm.

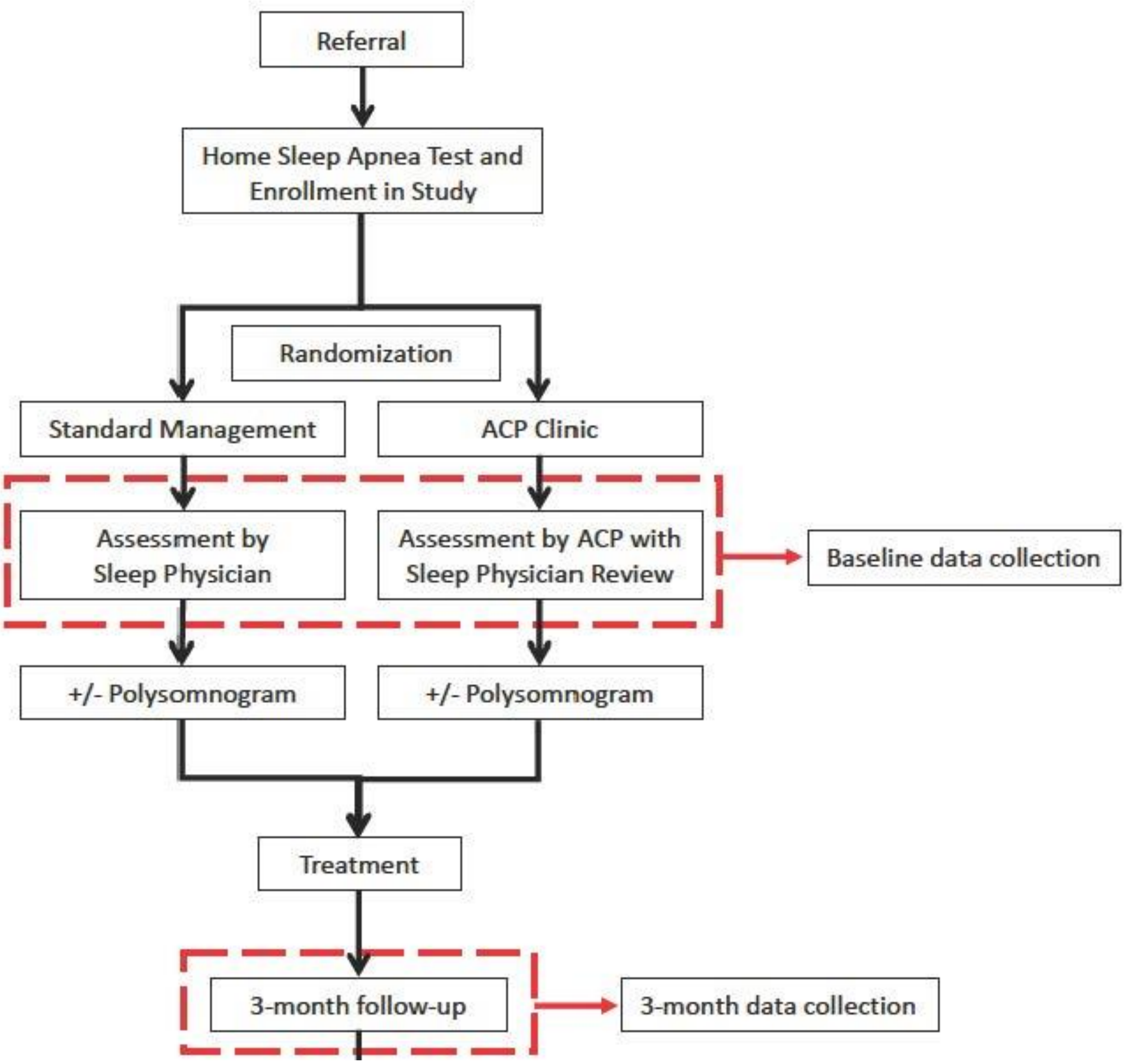

© 2020 Thornton CS et al. *JAMA Network Open.*
