# Supplementary material for: Effects of Wait Times on Treatment Adherence and Clinical Outcomes in Patients With Severe Sleep-Disordered Breathing: A Secondary Analysis of a Noninferiority Randomized Clinical Trial
Source: JAMA Netw Open. 2020 Apr 20;3(4):e203088. doi: 10.1001/jamanetworkopen.2020.3088 (PMC7171552; doi:10.1001/jamanetworkopen.2020.3088)
Supplement: Supplement 3. — Data Sharing Statement [file jamanetwopen-3-e203088-s003.pdf]

Thornton CS, Tsai WH, Santana, MJ, et al. Effects of wait times on treatment adherence and clinical outcomes in patients with severe sleep-disordered breathing: a secondary analysis of a noninferiority randomized clinical trial. *JAMA Netw Open*. 2020;3(4):e203088. doi:10.1001/jamanetworkopen.2020.3088

Data Sharing Statement

1. Will individual participant data be available (including data dictionaries): Yes.

2. What data in particular will be shared? Individual participant data that underlie the results reported in this article, after deidentification (text, tables, figures and appendices).

3. What other documents will be available? Study protocol, informed consent form.

4. When will data be available? Beginning 6 months and ending 3 years following article publication.

5. With whom? Researchers who provide a methodologically sound proposal.

6. For what types of analyses? To achieve aims in the approved proposal.

7. By what mechanism will data be made available? Proposals should be directed to Sachin.pendharkar@ucalgary.ca. To gain access, data requesters will need to sign a data access agreement.
